# Structured vulnerability: nationality stratifies wages, working conditions, and labor rights among migrant workers in Malaysian oil palm plantations

**DOI:** 10.3389/fsoc.2026.1865798

**Published:** 2026-08-19

**Authors:** Pafun Nilsawas Duhamel, Pasakorn Thammachote, Karh Chin Hoo

**Affiliations:** 1 School of Political Science and Public Administration, Walailak University, Nakhon Si Thammarat, Thailand; 2 Center of Geosocial and Cultural Research for Sustainable Development, Walailak University, Nakhon Si Thammarat, Thailand; 3 Department of Agricultural and Resource Economics, Faculty of Economics, Kasetsart University, Bangkok, Thailand; 4 Chung Hua High School, Seremban, Negeri Sembilan, Malaysia

**Keywords:** Bangladeshi migrant worker, Indonesian migrant worker, labor rights, Malaysian oil palm plantations, migrant worker, Thai migrant worker

## Abstract

Malaysia’s palm oil industry, the world’s second-largest, depends on migrant labor, with foreign workers making up 80% of its plantation workforce. Despite well-documented labor exploitation, research has typically focused on single nationalities, overlooked the interplay of structural and individual factors, and largely excluded Thai workers from comparative analyses. This study addresses these gaps by comparing the experiences of Thai, Indonesian, and Bangladeshi migrants on plantations along the Thai-Malaysian border. The study uses in-depth interviews, focus group discussions, field observations, and NGO input. A four-dimensional framework—structural, institutional, social, and individual—guides the analysis. Findings reveal that nationality-based vulnerability is generated through the interaction of global commodity chain pressures, governance failures, social network structures, and individual constraints. While all three groups occupy precarious, second-tier positions in the labor market, important differences emerge: Bangladeshi workers are concentrated in the highest-risk roles, earning less than 900 MYR per month and burdened by heavy employment debts; Indonesians fill a wider variety of jobs with moderate debts and earnings ranging from 600–1,300 MYR; Thai workers, mostly near the border, have lower debt and greater mobility, but remain highly vulnerable due to their undocumented status. Contrary to expectations from formal bilateral agreements, most Thai migrants enter Malaysia through informal networks, leaving them outside all institutional protection. Theoretically, the study introduces the concept of infrastructure shortcomings to capture how exclusion from regulatory frameworks—not just their distortion—creates risk for migrants, expanding Sunam’s notion of unstable infrastructure. The paper also develops the idea of secondary stratification within multi-national labor markets and distinguishes between freedom of exit and freedom of expression as distinct political dimensions. These contributions highlight the need for policy reforms that address both specific vulnerabilities and overarching structural barriers: employer-tied work permits, exclusion from union protections, and the institutional invisibility of undocumented migrants.

## 1 Introduction

Global palm oil production reached approximately 78 million metric tons in 2022–23 and remained at a comparable level of approximately 77.3 million metric tons in 2023–24, with Indonesia and Malaysia combined accounting for about 85% of the global supply (USDA Foreign Agricultural Service, 2024). This sustained scale of production comes with an increasing reliance on migrant labor, particularly in harvesting and plantation maintenance stages, which remain resistant to mechanization. Malaysia, the world’s second-largest palm oil producer, generated total palm oil export revenue of 109.39 billion Malaysian ringgit (approximately US$23.2 billion) in 2024, a 15.2% increase from 2023, and produced approximately 18.8 million metric tons of crude palm oil (CPO) in the same year (Malaysian Palm Oil Board [MPOB], 2024). Palm oil accounts for approximately 2.4% of Malaysia’s gross domestic product (GDP), underscoring its critical importance to the country’s economy (Statista, 2024).

The labor structure supporting this productivity is largely composed of migrant workers. As of 2022, approximately 382,000 workers were employed in Malaysia’s oil palm plantations, of whom about 80% were foreigners (Statista, 2023; MPOB, 2021). The industry faces a persistent and economically significant labor shortage. As of February 2024, Malaysia’s Minister of Crops and Commodities reported a shortage of approximately 40,000 palm oil workers, equivalent to a loss of approximately 7.9 billion Malaysian ringgit in annual export value (New Straits Times, 2024). The Malaysian Landowners’ Association reported that as of September 2022, there were 150,608 foreign workers employed in the agricultural sector, significantly exceeding the recommended labor-to-land ratio for sustainable, unmechanized operations across 2.6 million hectares of cultivated land, which is 1:14. A ratio of approximately 1:17 indicates that the agricultural sector is operating at around 80% of its required labor capacity (GAPKI, 2022; U.S. Department of Labor, 2021). This structural labor gap has historically been filled primarily by migrant workers from Indonesia, Bangladesh, Thailand, India, and Nepal, who took on physically demanding jobs that Malaysians consistently refused due to difficult working conditions, remote locations, and relatively low wages.

Despite the industry’s economic significance and heavy reliance on migrant labor, existing research reveals three significant limitations. First, research on labor migration from palm oil plantations in Southeast Asia remains fragmented, with Indonesian workers historically receiving the most academic attention due to their large numbers (Ford, 2006; Cramb and Curry, 2012). Bangladeshi workers appear primarily in literature on forced labor and human trafficking, focusing on cases of extreme exploitation (Fair Labor Association, 2018; The Arakan Project, 2015; Ford et al., 2012), and Thai workers—despite Thailand being the world’s third-largest palm oil producer with a projected output of 3.6 million metric tons in 2023–24—are virtually absent from the literature on palm oil plantation labor migration. This nationalistic bias hinders a systematic understanding of how migration regulatory structures contribute to differing vulnerabilities among workers sharing the same secondary labor markets.

Second, theoretical segregation is characteristic of studies on the exploitation of agricultural labor and migration. Labor market segmentation theory (Piore, 1979) explains why migrants are pushed into low-status, high-risk agricultural roles, but it does not account for how the migrant population itself is further subdivided by nationality, gender, and legal status. The unstable infrastructure framework (Sunam, 2022) demonstrates that systemic management can create positive risk, but this requires empirical evidence in the context of geographically isolated, employer-controlled farms. The political framework of unfreedom (Li, 2017a,b) reveals the use of a mix of freedom and unfreedom in agricultural labor systems, but its comparative application across different origin–destination migration routes remains limited.

Third, and most practically, the working conditions of migrant workers in Malaysia’s palm oil plantations have been documented as involving serious labor rights abuses that persist despite existing legal frameworks. The U.S. Customs and Border Protection (CBP) injunction against the release of palm oil produced by the Sime Darby Plantation in 2020 and subsequent enforcement actions by the U.S. Department of Labor highlighted indicators of systemic forced labor, including passport confiscation, wage deductions, and debt in the sector (Crowley, 2020; U.S. Department of Labor, 2021). Malaysia’s National Plan of Action on Forced Labor (NAPFL) 2021–2025, while demonstrating significant policy commitment, shows limited progress in law enforcement in the palm oil sector (Tan, 2025). The labor rights situation in this sector is further exacerbated by the job placement debt crisis affecting Bangladeshi workers. The cost of migration per worker is projected to be US$4,400–US$6,600 in 2023–2024, more than double the pre-2015 level and among the highest costs recorded globally (Bloomberg, 2026; Freedom United, 2026).

This research examines the three aforementioned limitations by conducting a systematic comparative analysis among Thai, Indonesian, and Bangladeshi migrant workers employed in oil palm plantations in the Thai–Malaysian border region of Perlis and northern Kedah. A four-dimensional analytical framework is used, integrating structural (global commodity chains and labor market segmentation), institutional (migration regulation and labor standards), social (migration networks and community integration), and individual (human capital, debt, and health) dimensions. The research investigates how macroeconomic structural pressures limit institutional capacity, how institutional organization controls migration drivers, how social networks determine recruitment and support, and how individuals experience the cumulative effects of cross-level dynamics. This research is particularly important in providing methodological and empirical evidence regarding undocumented Thai migrant workers, whose experiences are systematically missing from comparative literature on oil palm plantation workers.

## 2 Research objectives

This study analyses the working conditions, wages, and labor rights of Thai, Indonesian, and Bangladeshi migrant workers in Malaysian oil palm plantations through a four-dimensional framework (structural, institutional, social, and individual). The objectives are:

1. Examine how global and local economic structures shape migrant workers’ roles, wage hierarchies, and work assignments.
2. Analyze how migration policies and labor laws affect working conditions and vulnerabilities among different nationality groups.
3. Investigate the role of social networks in recruitment, integration, and access to support systems.
4. Assess how individual factors—such as skills, debts, and health—interact with broader structural, institutional, and social influences.
5. Integrate findings to identify both shared and nationality-specific challenges and offer policy recommendations to address exploitation in the industry.

## 3 Review literature

### 3.1 Migrant labor in southeast Asian plantation

The reliance on migrant labor in agricultural plantations across Southeast Asia is widely documented (Lindquist, 2010; Castles and Miller, 2009). Malaysia’s palm oil industry is one example of this structural dependence. As of 2022, approximately 382,000 workers were employed in Malaysia’s oil palm plantations. Foreign nationals comprise approximately 80% of the total workforce (Statista, 2023; MPOB, 2021). This proportion has remained consistently high over the past two decades. Data from MPOB show that foreign workers accounted for 69% of the plantation labor force in 2010, rising to 76.5% in 2012, 78% in 2015, and remaining around 80% in the early 2020s (Abdullah et al., 2011; Azman, 2015; MPOB, 2021). The ongoing labor shortage—officially estimated at 40,000 as of February 2024—results in an economic loss of approximately 7.9 billion Malaysian ringgit per year from unharvested fresh palm fruit (New Straits Times, 2024; Crowley, 2020), which reflects the structural unattractiveness of agricultural employment for Malaysians and the disruption to established migrant labor supply chains following the COVID-19 pandemic.

Academic research indicates that migrant agricultural workers face distinctly different risks due to the specific institutional structures of their employment. These include remote geographical locations that cut them off from legal and civil society resources; employer-controlled housing that allows for comprehensive surveillance and restrictions on movement (Caxaj and Cohen, 2019; Jones et al., 2024); industry-specific legal exclusions, particularly the prohibition of agricultural workers from joining trade unions under Section 26A of the Malaysian Trade Unions Act 1959; and restricted movement due to the employer-controlled work permit system. These conditions combine to create an environment conducive to labor exploitation, including unpaid wages, debt, and confiscation of travel documents. And physical and mental abuse (Verité, 2016; IOM, 2014; Amnesty International, 2010). The U.S. Customs and Border Protection (US CBP) withholding orders issued to a major Malaysian palm oil company during 2020–2021 internationally confirm the prevalence of indicators of forced labor in this sector (US CBP, 2020; RSPO, 2020).

### 3.2 Employment practices and debt burden

Labor recruitment mechanisms serve as the primary institutional gateway for migrant workers entering exploitative on-farm employment systems. Comparative research reveals significant differences in recruitment channels, cost structures, and debt burdens across countries of origin, which directly affect labor vulnerability.

In contrast to Bangladeshi and Thai workers, Indonesian workers use both officially authorized agencies and informal networks (including pengguru, or illegitimate border facilitators) for employment. Systematic collusion between these facilitators and Malaysian immigration officials exists (Ford and Lyons, 2011; Kaur, 2010). The Indonesian government’s free migration policy for crop and oil palm sectors, implemented under the 2017 Indonesian Migrant Workers Protection Act and renewed in 2022, has shifted official employment costs to Malaysian employers, although enforcement gaps persist (Hall, 2024). Informal immigration remains common, with border facilitation costs historically ranging from 130 MYR at official crossings to over 1,000 MYR via pengguru, highlighting stark cost differences compared to other nationalities (Ford, 2006).

Unlike Indonesian or Thai workers, Bangladeshi workers face far greater recruitment costs and debt burdens. The dominance of Bestinet’s digital recruitment system and brokerage fee structures since 2015 has driven costs from the official US$650 to US$4,400–US$6,600 per worker by 2023–2024, more than double pre-2015 rates and dauntingly higher than for other nationalities (Bloomberg, 2026; International Organization for Migration, 2023). In 2023, Malaysia’s Prime Minister called the system “modern-day slavery”. Over 70% of Bangladeshi workers spend at least half their wages on debt, often holding multiple loans with interest rates up to 30% (Associated Press, 2025; Tan, 2025). About 38% borrow to pay job placement fees—nearly double official limits—which, combined with average monthly deductions of 400 ringgit for costs, contrasts sharply with the lower financial burden on Indonesian and Thai workers. Crackdowns in 2024 led to the arrest of dozens of Bangladeshi placement agents for money laundering and trafficking, underscoring a uniquely severe context (Bloomberg, 2026).

For Thai workers, finding work on farms in Malaysia mostly occurs through informal cross-border social networks rather than through authorized recruitment agencies. This aligns with the chain-like migration dynamics characteristic of labor flow in border areas (Papademetriou et al., 2010). Research on Thai workers returning from farms in Malaysia (including studies from the IMT-GT border labor context) confirms that undocumented status is the most common form of immigration, driven more by social job placement through family and community networks than through formal bilateral channels, despite the existing Memorandum of Understanding on labor cooperation between Thailand and Malaysia (Crowley, 2020; Duhamel and Hoo, 2026). Fees for informal cross-border contractors are approximately 1,000–3,000 baht (approximately 120-360 Malaysian ringgit), significantly lower than the recruitment costs for Indonesian or Bangladeshi workers, and do not create a significant debt burden for Thai workers.

### 3.3 Employment conditions and labor protection violations

Substandard working conditions in Malaysia’s oil palm plantations have been comprehensively documented using various research methodologies and reporting frameworks. The structure of the sector gives rise to consistent patterns, including: wages below the national minimum wage (currently 1,700 MYR per month), effective February 2025 for employers with five or more employees, up from 1,500 MYR under the 2024 Minimum Wage Order published in the December 2024 Gazette; excessive working hours; a piecework wage structure that creates income volatility and incentivizes unproductive attendance while avoiding hourly wage protection; inadequate or non-existent personal protective equipment for pesticide, fertilizer, and machinery handling; employer-controlled housing with documented deficiencies in sanitation, water supply, and medical access; and systemic barriers to formal grievance mechanisms stemming from bureaucratic status, language barriers, geographical isolation, and legal prohibitions on unionization for plantation workers (Amnesty International, 2010; Anti-Slavery International, 2006; Puder, 2019; International Labour Organization (ILO), 2021).

Performance-based wage systems deserve special consideration as they are deeply ingrained mechanisms for wage suppression and labor control. Under performance-based wage agreements used in Malaysia’s oil palm plantations, workers are paid per ton of fresh fruit (FFB) harvested or per task completed, rather than per hour worked. This system creates systematic income volatility, linked to seasonal yield variations, worker assignments, terrain, and tree height—factors beyond the workers’ control. Simultaneously, it incentivizes workers to neglect rest, medical care, and safety measures to maximize daily productivity. Studies among workers of three nationalities consistently show that performance-based wages fall short of the national minimum wage, a pattern exacerbated by employer deductions for housing and utilities (200–400 MYR per month), as documented by International Organization for Migration (2023). The 2024 Minimum Wage Directive explicitly states that workers paid solely on the basis of volume, weight, work, travel, or commissions must receive a minimum wage of 1,700 MYR per month (Lexology, 2025). However, enforcement in remote plantations remains significantly inadequate.

Malaysia’s legal framework—the Employment Act 1955, the Minimum Housing and Work Facilities Standards Act 1990, the Anti-Trafficking in Persons and Immigration Act 2007, and the National Action Plan on Forced Labor 2021–2025—provides a nominal comprehensive regulatory structure. However, in practice, gaps between legal provisions and reality on plantations are widely documented. Labor inspections in remote farm areas are infrequent; undocumented workers lack access to formal complaint mechanisms without risking deportation; and the employer-tie work permit system prevents workers from changing employers without losing their legal right to remain in Malaysia—a condition recognized by the International Labor Organization as an indicator of forced labor (International Labour Organization, 2012; International Labour Organization (ILO), 2021; Nah, 2012).

### 3.4 Digital technology and labor regulation

Recent research examines how digital innovation may enhance labor governance and protection across complex and geographically distributed labor supply chains. Technologies under scrutiny include blockchain-based supply chain tracking systems, mobile payroll and complaint platforms, digital labor identity and documentation verification systems, and data analytics for labor audit compliance (Aneesh, 2009; Martin, 2017). Malaysia’s palm oil industry’s involvement in digital labor governance is most evident in the Bestinet digital platform controversy, which centralized the recruitment of Bangladeshi workers under a monopolistic digital system designed to reduce fraud but, in practice, created conditions for systematic fee increases and exploitation by influential groups. This case study shows how digital infrastructure can amplify exploitative recruitment structures rather than mitigate them (Bloomberg, 2026). Industry involvement in the Malaysian Sustainable Palm Oil Certification Program (MSPO) and the Roundtable on Sustainable Palm Oil (RSPO) framework, both of which have labor rights standards requiring digital verification documentation, has resulted in limited improvements in working conditions, particularly for undocumented migrant workers. This is outside the official labor registry and cannot be verified during the certification process.

## 4 Analytical framework

This research employs a multi-level analytical framework that integrates institutional and individual levels to describe the conditions, experiences, and outcomes of migrant workers in the palm oil industry. Comparative analysis is used to capture differences between countries or regions due to governance systems, labor market structures, and socio-cultural backgrounds (Bloemraad and Wright, 2014; Castles et al., 2020).

### 4.1 The structural dimension

#### 4.1.1 The global political economy of plantation labor

This conceptual framework positions migrant plantation workers within the global supply chain and the international labor mobility system. The plantation sector (e.g., palm oil, rubber, fruit, and sugar) often relies on low-skilled migrant workers due to structural labor shortages. Seasonality and cost-competitive production (Rigg, 2019). Global demand for cheap agricultural commodities creates a divide between citizens and migrant workers, affecting wages, jobs, and vulnerability to oppression.

#### 4.1.2 The labor market segmentation theory helps explain why migrants are being replaced by low-status and dangerous crop workers

The bi-sectoral labor market, consisting of the first (stable and high-wage) and second (unstable and low-wage) sectors, has systematically routed migrants to the second sector (Piore, 1979). This structural segmentation determines the working conditions, mobility opportunities, and risks of unstable employment.

### 4.2 Institutional dimensions and policies

#### 4.2.1 Migration regulations and legal status

Institutional frameworks shape immigration pathways, legal protection, and the risk of illegal employment for migrants (Koser, 2016). These frameworks include migration policies, recruitment rules, and bilateral agreements. Comparative analyses examine how national regulatory systems differ, such as employer-defined visas, work permits, and enforcement approaches. These differences affect the vulnerability or empowerment of agricultural workers.

#### 4.2.2 Labor standards, law enforcement, and employer practices

National labor laws and occupational safety standards play a crucial role in regulating working hours, contract fairness, wage protection, and grievance mechanisms. Weak law enforcement on rural farms increases the risk of forced labor, debt burden, and exposure to hazards (International Labour Organization (ILO), 2021). Employer practices (e.g., subcontracting, labor brokers) create institutional intermediaries that can exacerbate exploitation.

### 4.3 Social and community dimensions

#### 4.3.1 Social network theory

Immigrant networks shape migration decisions, employment channels, job placements, and support systems. Networks reduce migration costs and risks. However, they may also lock workers into utilitarian arrangements through employment or relative debt (Massey et al., 1993). Comparative analyses examine differences in network structure across countries or immigrant groups.

4.3.2 Community Integration and Social Participation: The level of social integration in the local community affects migrant wellbeing, access to services, and long-term livelihood. Factors such as language barriers, practices, and cultural norms define the scope of social participation or exclusion (Ager and Strang, 2008).

### 4.4 Individual dimensions

Human capital theory and human capital utilization skills show that immigrants’ education and agricultural experience influence employment and income patterns (Becker, 1993). In large-scale farm environments, these skills may not be fully utilized. Sector limitations or employer requirements for low-skilled labor can restrict their use.

Personal experiences of welfare, health, and safety outcomes range from work-related injuries to psychological stress. These outcomes result from the interaction between structural and practical constraints in the workplace. Health varies depending on chemical exposure, labor volume, and living conditions (UNDP, 2022).

## 5 Research methodology

This research employs a qualitative comparative case study design informed by a phenomenological sensibility. The phenomenological orientation justifies close attention to the lived experiences, subjective meanings, and social and cultural contexts of migrant workers, while the comparative case study strategy enables a systematic analysis of similarities and differences among three nationality groups—Thai, Indonesian, and Bangladeshi—employed in oil palm plantations along the Thai–Malaysian border. The study does not apply a single-tradition phenomenological method, such as interpretative phenomenological analysis or Giorgi’s descriptive phenomenological method; rather, thematic analysis is used to construct themes that are then systematically compared across the three nationality groups (see Section 5.5). This design is particularly suitable given the sensitive and complex nature of the migrant worker situation: extensive, contextually grounded data are necessary to reveal structural inequalities, wage disparities, and labor rights violations, which cannot be adequately captured by quantitative tools alone.

### 5.1 Study area

Fieldwork was conducted in oil palm plantations located in the Thai-Malaysian border region, particularly in the states of Perlis (Kangar District, bordering Satun Province, Thailand) and northern Kedah (Padang Besar and Changlun Districts, bordering Songkhla and Satun Provinces, Thailand). These areas were specifically chosen for four reasons: (1) they are important oil palm producing areas with large populations from the three target nationalities; (2) their border location facilitates the movement of both legal and illegal labor; (3) civil society organizations focused on labor rights operate in the region; and (4) researchers had access to established networks of migrant worker communities.

### 5.2 Participants and sampling

#### 5.2.1 Primary informants

This study involved 30 migrant workers, comprising 10 Thais, 10 Indonesians, and 10 Bangladeshis. Each subgroup consisted of 6 men and 4 women (the Bangladeshi subgroup instead comprised 7 men and 3 women, reflecting the overwhelmingly male composition of the Bangladeshi oil palm plantation workforce), aged between 22 and 50, with diverse educational backgrounds, documentation statuses, and recruitment paths. Inclusion criteria required participants to: (a) have at least 6 months of work experience in oil palm plantations in the study area; (b) be 18 years of age or older; (c) be able to communicate in their native language or have basic proficiency in Malay or English; and (d) provide voluntary and fully informed consent. These criteria ensured that participants had sufficient work experience to provide material information while maintaining ethical standards regarding their ability to provide informed consent.

#### 5.2.2 Secondary informants

Four staff members from two labor rights NGOs operating in Malaysia’s border region were interviewed as secondary informants. One NGO has been providing legal aid, shelter, and support to migrants and victims of trafficking. The other NGO focuses on labor justice, union support, and a regional support network spanning Thailand, Indonesia, and Bangladesh. Both organizations have documented case studies and field data on working conditions in the study areas that supplement direct worker accounts. Where accessible, additional informants were interviewed, including plantation managers (*n* = 2) and local labor inspectors (*n* = 1) to obtain perspectives from employers and a regulatory perspective.

### 5.3 Sampling strategy

Purposive sampling was used as the primary strategy to ensure participants met eligibility criteria and reflected diversity in gender, age, document status, and recruitment channels. This was supplemented by chain sampling, where the first group of participants introduced the next group of contacts within their social networks. Reaching labor participants was coordinated through NGOs’ field staff, who introduced the research team to community leaders, thereby minimizing suspicion and reducing response bias due to unfamiliarity. Sampling continued until topic saturation was reached in each nationality group.

### 5.4 Data collection

#### 5.4.1 Instruments

Three main data collection tools are used.

1) Semi-structured in-depth interview guides

The semi-structured in-depth interview for workers covers nine topics: background and motivation before relocation; recruitment process and related costs; debt burden; working conditions and wages; living conditions and freedom of movement; occupational health and safety; labor rights awareness and violations; social networks; and future plans. The interview for Non-Governmental Organizations focuses on the organization’s mission, the situation of migrant workers in border areas, the recruitment structure, the legal framework, access to justice, and policy suggestions. Each interview was conducted in the participants’ native language with a trained interpreter and lasted 90–120 min.

2) Focus group discussion (FGD)

Three group discussions were conducted, separated by nationality (Thailand, Indonesia, and Bangladesh; 8–10 participants per group). FGD participants were drawn from the same 30 workers who completed the individual interviews (8–10 of the 10 participants per nationality); this deliberate overlap allowed the group setting to surface collective and relational dimensions that complemented, and were triangulated against, the individual accounts, using a structured facilitation protocol. Discussion topics covered motivations for migration and recruitment experiences, daily work life, labor rights violations, inter-ethnic relations, and collaborative recommendations for improvement. Participatory activities (anonymous cost mapping, group storytelling, priority voting) were integrated to generate information not readily available through individual interviews. Each group consultation lasted 120–150 min, and audio and video recordings were made with participants’ consent.

3) Structured observation protocol

Both participatory and non-participatory observations were conducted in five areas. These included: (1) the field working environment (terrain, equipment, chemical handling, and personal protective equipment), (2) interactions between workers and supervisors, (3) housing (density, sanitation, utilities), (4) social life and inter-ethnic dynamics, and (5) indicators of vulnerability (limited mobility, signs of physical or mental distress). Daily field notes, photographs (with informed consent), and reflective memos were kept throughout fieldwork.

#### 5.4.2 Data collection procedure

To ensure systematic implementation, the data collection process was divided into five distinct phases, each building upon the previous stage.

Phase 1 (Month 1: Preparation and Networking) consisted of coordinating with NGO staff, negotiating access to the cultivation area, and recruiting and training three research assistants/translators. Consisting of native-speaking Thai, Bahasa Indonesian, and Bengali translators, each with original experience in campaigning for migrant workers, the training courses cover research ethics, in-depth interview techniques, and field observations. Cultural sensitivities and emergency procedures were also tested using instruments with 9 workers (3 per ethnicity), and the manual was adapted based on the results of the experiment. These 9 pilot participants were recruited separately for instrument testing and are not included in the 30 main study participants.

Phase 2 (Month 2: NGO Staff Interviews) consisted of four interviews with NGOs staff at their offices, focusing on providing background information and introducing the communities.

Phase 3 (Months 3–5: Labor Interviews and Observations) involved ongoing in-depth interviews with all 30 participating workers in both locations, along with both participatory and non-participatory observations. Follow-up interviews with 5–10 participants were conducted in Month 5 to clarify any emerging issues; these follow-up interviews constituted the concluding step of Phase 3.

Phase 4 (Month 6: focus group discussions) comprised three focus group discussions held over a weekend at a neutral community venue (an assembly hall/community pavilion), facilitated by the principal investigator, with two assistants and an interpreter.

Phase 5 (Months 7–10: Secondary Data Collection, Transcription, and Analysis) covered document collection (NGO reports, labor statistics, policy documents, media coverage), transcription and translation, data organization, and systematic analysis.

All interviews and group discussions were digitally recorded using two recorders for backup. To ensure data integrity, all files were backed up daily to encrypted external storage and cloud servers. Immediately after data entry, personal identifiers were replaced with alphanumeric codes (e.g., TH-01, ID-05, BD-10), thereby enhancing participant confidentiality throughout the process.

### 5.5 Data analysis

All audio recordings were meticulously transcribed into their native languages before being translated into English by individual interpreters and reviewed by language experts. The original language recordings were preserved alongside the translations to maintain semantic accuracy. Nonverbal cues, emotional tone, and contextual observations were enclosed in parentheses to support interpretation accuracy. Qualitative data analysis followed Braun and Clarke’s (2006) six-step thematic analysis framework, using NVivo 14.

Step 1 (Familiarization) involved rereading all 30 individual transcripts, 3 group transcripts, NGO interview recordings, and field notes.

Step 2 (Initial Coding) employed data-driven open coding, supplemented with theoretically informed coding, prioritizing *in vivo* labels that closely resembled participants’ own expressions; this process generated 114 initial codes.

Step 3 (Theme Development) clustered these open codes into candidate themes and sub-themes on the basis of shared analytical meaning rather than surface wording, with the clustering process documented through successive thematic maps.

Step 4 (Theme Review) re-examined the candidate themes at two levels—first against the coded extracts and then against the full dataset—to confirm internal coherence, distinctiveness, and comprehensive coverage; candidate themes that overlapped analytically were merged, and those insufficiently grounded in the data were discarded. Through this iterative process, the 114 initial codes were consolidated into 4 final themes and 14 sub-themes.

Step 5 (Defining and Naming Themes) produced concise, analytically precise theme names together with detailed definitions specifying the scope and boundary of each theme.

Step 6 (Reporting) integrated the thematic narratives with selected participant quotations and theoretical interpretation. The final themes reported in Section 6 map directly onto the four dimensions of the analytical framework.

Cross-case comparative analysis was employed, beginning with an analysis of data within each nationality group (in-case analysis) before systematically comparing patterns between groups using thematic matrices. Convergence and divergence patterns were identified and interpreted in reference to structural factors, including recruitment channels, bilateral policy frameworks, social capital, and employer discrimination. Descriptive analysis was applied to six to nine selected life stories (two to three per nationality) to illustrate the temporal structure of migration experiences and the subjective meanings workers assign to their situations. The analysis was also anchored within three theoretical frameworks: labor market segmentation theory (Piore, 1979), the unstable infrastructure framework (Sunam, 2022), and the politics of unfreedom (Li, 2017a,b), supplemented by a correlational perspective considering the interactions of nationality, gender, class, and documented status.

### 5.6 Data verification

The rigor was established according to the four criteria of reliability proposed by Lincoln and Guba (1985):

Reliability: continuous engagement (6 months of fieldwork in both areas), which fostered deep relationships and contextual understanding; ongoing observation under diverse times and conditions, which strengthened empirical data; the use of methodological triangulation, integrating data sources from interviews, group discussions, observations, and documents; the application of data triangulation, verifying data accuracy through workers’ narratives, institutional knowledge of NGOs, and direct observation; and investigator triangulation, achieved through coding and joint interpretation by the research team. Member-checking involved 10–15 participants who reviewed preliminary analytical summaries, and their feedback refined the findings. External experts, including migration scholars and NGO specialists, provided conclusions.

Transferability: This research provides comprehensive details of the research context, participant characteristics, and data collection procedures, enabling readers to assess the suitability of the research findings to comparable contexts.

Dependability: detailed verification records are provided, encompassing raw data (audio files, field notes, photographs), data reduction products (transcripts, encoding frameworks, memos), analysis results (thematic maps, matrices), and process logs documenting methodological decisions and reflective responses. External verification by independent qualitative researchers confirmed the consistency of the analytical procedures.

Confirmability: The reliability was maintained by documenting self-reflection throughout data collection and analysis, including the researcher’s stance, potential biases, emotional responses to sensitive content, and the dynamic power dynamics between the researcher and participants. External expert verification confirmed that the interpretive conclusions were based on empirical evidence rather than on the researchers’ speculation.

### 5.7 Ethical considerations

This study was approved by the Walailak University Human Research Ethics Committee (WUEC-26-059-01) prior to data collection. All participation was voluntary, and informed consent was obtained in writing or via audio recording (for participants with limited literacy) after the study’s purpose, procedure, risks, benefits, and right to unconditional withdrawal were fully explained. Confidentiality was protected through the use of pseudonyms and secure data storage. No farm names or identifiable geographical details were reported. The principle of harmlessness was practiced by: selecting private interview locations outside the workplace to prevent employer surveillance; avoiding questions that might cause psychological harm; providing guidance on accessing psychological support when distress was observed; and a strict policy of non-disclosure of document status to authorities. Undocumented workers were not pressured to disclose their legal status, and the research team maintained clear boundaries with regard to law enforcement agencies.

### 5.8 Methodological limitations

This research acknowledges several methodological limitations that should be considered when interpreting the study results.

Geographic scope and sampling: This study focused on oil palm plantations in Perlis and northern Kedah near the Thai-Malaysian border. Findings may not extend to other regions, such as Sabah, Sarawak, or other areas in Peninsular Malaysia, which differ in size, labor makeup, and regulations. A sample of 30, suitable for qualitative research, may not represent the full range of migrant worker experiences. Chain sampling may also cause similarity bias, as socially connected participants may share similar experiences and perspectives.

Limitations in access and self-selection: Facilitating access through NGO networks may result in a sample of workers with higher awareness of labor rights than the general farm worker population. Workers facing the most severe restrictions, including those in situations similar to forced labor, may be unable or unwilling to participate, potentially leading to underestimations of the most serious abuses. Fear of job loss or deportation among undocumented migrant workers may also result in underreporting of working conditions.

Language and cultural mediation: Even with bilingual research assistants, some meaning may be lost, especially for cultural expressions or emotions. Cultural norms about discussing personal or professional hardship may also reduce honest responses.

Social desirability and memory bias can lead participants to adjust their answers to align with the researcher’s expectations (social desirability bias), especially on contentious topics such as illegal status or labor rights violations. Additionally, early-stage records on recruitment costs and working conditions may also be influenced by memory bias.

Cross-sectional design: The cross-sectional methodology prevented tracking changes in conditions and vulnerabilities over time or across seasonal labor cycles. The findings provide an overview of participants’ experiences during a set period in 2024–2025.

Contextual and policy volatility. Rapid changes in Malaysia’s immigration and labor policies, bilateral memoranda of understanding, and the dynamics of global commodity markets may limit the time relevance of the research findings. Researchers should consider the policy environment in effect at the time of data collection when interpreting the results.

These limitations have been addressed through validation methods, expanded field research, participant verification, and transparent data reporting. Despite these limitations, this research provides grounded qualitative evidence on migrant labor conditions in strategically important yet underresearched border agricultural areas.

## 6 Findings

The global political economy of palm oil acts as a structural driver pushing migrant workers into secondary segments of the Malaysian palm oil labor market. Within the commodity chain dominated by large multinational agribusinesses, value is disproportionately distributed among processors, traders, and retailers in consuming countries, while the most labor-intensive and least secure jobs are shifted to low-wage migrant workers. This segmentation occurs simultaneously on multiple axes: nationality, gender, document status, and length of stay, creating a stratified division of labor where vulnerability is concentrated at the bottom. Table 1 summarizes the comparative structural positions of the three groups.

**Table 1:** Comparative structural positioning of migrant worker nationality groups in Malaysian oil palm plantations.

Dimension	Thai workers	Indonesian workers	Bangladeshi workers
Structural push factor	Low Major palm producer itself; domestic labor alternative	Moderate ↓ Narrowing income gap; declining workforce share since 2012	High Severe labor surplus; per-capita income ~1/5 of Malaysia’s
Migration channel	Predominantly undocumented Informal cross-border via kin networks; no work permit	Dual channel Licensed agencies + informal pengguru; widespread undocumented entry	Nominally formal (G2G) G2G channel subverted by recruitment syndicate; inflated fees
Wage range (MYR)	~900 to 1,300 est. Piece-rate; below 1,500 minimum (fieldwork period)	600–1,300 (male) 800–1,000 (female) Below 1,500 minimum; deductions 200–400/month	<900 (gross) Highest wage theft; deductions 200–400/month
Recruitment debt (MYR)	Low ≈120–360; repaid within weeks	Moderate Formal: near zero (2022 MOU); informal: 1,000+	Extreme ≈20,000–30,000 actual vs. ≈ 3,000 mandated; loans up to 30% interest
Structural exit capacity	Moderate–High Proximity to Thailand; low debt; informal return feasible	Moderate Network options; return possible but costly	Low Debt makes exit catastrophic; 3–5 years’ income to clear

All monetary values in MYR; foreign-currency amounts converted at 2023/24 average rates (approximate 2023/24 averages: 1 USD ≈ 4.6 MYR; 1 MYR ≈ 7.7 THB, i.e., 1,000–3,000 THB ≈ 130–390 MYR). Geographic concentration, task assignment, the piece-rate system, and institutional protection status are described in the text.

### 6.1 Structural dimensions: labor market segmentation, wage hierarchy, and job assignment by nationality

Indonesian laborers have dominated palm oil plantations since independence, accounting for approximately 87% of field workers in 2012 (87% refers to the proportion of Indonesian field workers, which differs from the 76.5% figure in section 3.1, which represents the proportion of all foreign workers in the palm oil plantation workforce—these are different figures). They are largely from Javanese, Bugis, and Toraja ethnic groups. Their concentration reflects a long migration route to Sabah, the country’s main palm oil export destination (approximately 30%). However, this dominance is waning as Indonesia’s development narrows the GDP gap with Malaysia, weakening relative driving forces and gradually reducing the share of Indonesian labor. Nevertheless, this extensive migration network still provides Indonesian workers with access to a wide range of jobs, including harvesting, maintenance, and processing. Under the work-based wage system, when the minimum wage was 1,500 MYR per month (May 2022–January 2025), male harvesters earned approximately 600–1,300 MYR per month, and women performing tasks such as spraying, fertilizing, and fruit picking earned around 800–1,000 MYR per month, well below the minimum wage at the time. This is significantly lower than the minimum wage of 1,700 Malaysian Ringgit announced in February 2025. Furthermore, employers deduct approximately 200–400 Malaysian Ringgit per month for housing and utilities, further reducing net income (Tan, 2025; Verité, 2024; Bloomberg, 2026). This structural compression of income through deductions is detailed by an Indonesian harvester:

*“Because of our long-standing community networks here, many of us can actually get a wider range of skilled jobs in the mills and the fields. But honestly, the piece-rate system completely ruins our actual take-home pay. During peak harvesting seasons, my gross earnings might get close to the baseline. But then the management goes ahead and makes arbitrary monthly deductions of 300 to 400 MYR just for our crappy housing and electricity, so our net income drops big time. The cost of living in Malaysia has gone way up, but our piecework rates have been completely stuck.”*— (Transcript Identifier: IDN-INT-02, Male Harvester, Perlis).

Bangladeshi migrant workers are the fastest growing group, largely hired through government-to-government (G2G) channels with significant private sector involvement. Their situation reflects the convergence of a severe domestic labor surplus coupled with Malaysia’s demand for cheap and obedient labor, which is willing to accept conditions that stronger bilateral protections would reject. They are concentrated in the highest-risk jobs, including the manual transport and handling of fresh fruit, and those with high risks of pest infestations and accidents. Field data indicates their starting wages are generally below 900 MYR per month under the performance-based wage system, representing approximately 60% of the 1,500 MYR minimum wage, plus housing deductions of 200–400 MYR (Tan, 2025; Verité, 2024; Bloomberg, 2026). Debt repayment expenses exceed 50% of monthly net income for over 70% of workers, and they have the highest wage fraud rates of the three groups, with recorded debts ranging from US$4,400–US$6,600 while wages are below 900 MYR. Paying off the entire debt would require three to 5 years of cumulative income, which is more of a structural obligation than a temporary limitation. The severe occupational hazard and wage disparity faced by this cohort are highlighted in the following narrative:

*“They systematically funnel us into the most back-breaking and dangerous jobs on the estate, mostly hauling these heavy fresh fruit bunches by hand up and down steep hills. Even though we take on the highest-risk work, they purposely set our starting piece-rates lower than other groups, so we often end up making less than 900 MYR a month. But because we arrive here with massive recruitment debts to clear, we have absolutely no choice. We just have to accept these terrible rates and dangerous conditions without saying a word.”*— (Transcript Identifier: BGD-INT-05, Male General Worker, Perlis).

Thai workers face a drastically different status. Thailand’s status as the world’s third-largest palm oil producer (Crowley, 2020) significantly reduces the driving factors; namely, the availability of comparable agricultural jobs within the country reduces the wages employers can charge other groups. Therefore, Thai workers are concentrated in border states like Perlis, Kedah, and Kelantan, attracted by short-term income disparities and cross-border kinship networks, rather than being displaced due to severe economic hardship. The result is a geographically fragmented sub-market, distinct from Thailand’s larger agricultural labor market.

Thai workers are assigned narrower jobs than Indonesian workers, mostly harvesting and maintenance in small- to medium-sized palm plantations along the border, and are paid on a similar piecework system with comparable income volatility. Their wages appear slightly higher than those of Bangladeshi workers and comparable to Indonesian workers for equivalent positions, possibly reflecting the bargaining power gained from domestic alternatives: the proximity of the Thai labor market limits employer competition in setting wages, which is absent for Bangladeshi or Indonesian workers due to the high cost of returning to work or social stigma. However, these advantages—lower inbound debt and reduced risk of human trafficking—are also significant. And the options for leaving the country do not free Thai workers from the critical conditions of the secondary labor market: wages below the minimum wage, income insecurity from piecework, and hazardous working conditions found in all nationalities. The strategic deployment of geographic proximity as an economic safety valve is explained by a Thai border worker:

*“The main reason we still have a bit of bargaining power in these border plantations is simply because we are so close to the farming market back in Thailand. If a Malaysian boss tries to cut our piece-rates too low or forces us to work in dangerous conditions, we aren’t trapped here like the workers who come from far away. We can easily use our connections at the border to just head back home and work on plantations in Satun or Songkhla. That’s what forces the employers here to keep their wages a bit more competitive—they know they have to if they want to keep us around.”-* (Transcript Identifier: TH-FGD-01, Male Harvester, Perlis Border Zone).

### 6.2 Institutional dimension: migration governance, recruitment systems, and labor standard enforcement

Institutional frameworks—including bilateral agreements, national immigration and labor laws, recruitment oversight frameworks, and law enforcement capabilities—represent a second dimension in regulating working conditions and are used to limit or amplify structural market pressures. Evidence suggests that institutional frameworks across all three nationality groups have significant implementation gaps, systematically harming both workers and employers.

#### 6.2.1 Recruitment pathways and cost structures

Indonesian Labor: The channels for recruiting Indonesians are diverse, ranging from officially licensed entities to unofficial networks involving *pengguru* (illegal border facilitators). The official crossing of the border from Kalimantan to Sabah costs approximately 130 Malaysian Ringgit (MYR), while crossing through a *pengguru* costs from 1,000 MYR or more, with field evidence indicating recurring patterns of complicity with local immigration officers. The duality of this institution—where official and unofficial channels coexist—reflects the failure of the bilateral regulatory framework to enforce the use of regulated pathways. This dual recruitment framework and its financial implications are described by an Indonesian worker:

*“Many of us bypass the official channels because the government paperwork just takes way too long. Instead, we turn to the panggurus network to help us cross into Sabah. But going through this informal route jacks up our entry costs big time—we end up paying over 1,200 MYR compared to the tiny official fees. We pay this huge premium because we are told these fixers have already set things up with the border officials to make sure we cross without any trouble. But the thing is, it immediately leaves us completely broke and vulnerable the moment we arrive.”*— (Transcript Identifier: IDN-INT-05, Male General Worker, Perlis).

Bangladeshi Workers: Bangladeshi workers experience one of the most systematically disadvantaged job procurement systems in the world. Although the officially mandated recruitment fee remains approximately USD 650 per worker, documented total costs incurred by Bangladeshi workers—inclusive of broker fees, syndicate charges imposed through the Bestinet digital recruitment platform, and associated migration costs—ranged from USD 4,400 to USD 6,600 per worker in 2023–2024 (Bloomberg, 2026; Freedom United, 2026). The Bloomberg (2026) figures derive from an economy-wide investigation of Bangladesh–Malaysia recruitment (covering construction and other sectors) and are triangulated here with sector-specific estimates from International Organization for Migration (2023) for palm oil plantation labor. A 2024 memo prepared for Malaysian Prime Minister Anwar Ibrahim by the Madani Research Center found these costs to be more than double those paid before Bestinet’s involvement (Bloomberg, 2026). Malaysian Prime Minister Anwar Ibrahim publicly described the recruitment agent system as ‘modern slavery’ in 2023 (Tan, 2025; Bloomberg, 2026).

A 2024 study found that over 70% of Bangladeshi migrant workers in Malaysia had allocated at least half of their monthly wages to servicing recruitment debts, with most holding multiple concurrent loans at interest rates of up to 30% (Verité, 2024; Office of the United Nations High Commissioner for Human Rights, 2024). Furthermore, International Organization for Migration (2023) documented that approximately 38% of migrant workers in oil palm plantations specifically took out loans to cover recruitment costs, which were already almost double the mandated ceiling, with employer deductions for accommodation, utilities, and food adding approximately 400 MYR per month to workers’ financial burden. The long-term financial dependency dictated by this recruitment structure is illustrated by a Bangladeshi laborer:

*“To be honest, the whole system to get here is just a trap to keep us in debt, not a real way to find a job. By the time you pay all those layers of agents, sub-agents, and the fees on that digital platform, you are looking at around USD 5,500 just to secure this spot. Right now, more than 60% of my monthly piece-rate earnings goes straight to paying off informal lenders who charge a crazy 25% interest rate. With this financial burden, I have absolutely no choice or freedom to go anywhere else. I’m completely stuck on this plantation because if I walk away or speak up against their wage cuts, it would completely ruin my family back home.”*— (Transcript Identifier: BGD-INT-04, Male General Laborer, Perlis).

Thai Workers: Contrary to the concept of formal bilateral cooperation channels outlined in the Memorandum of Understanding on Labor Cooperation between Thailand and Malaysia, empirical evidence from the context of border palm plantations indicates that the dominant pattern of entry for Thai workers in these plantations is illegal, undocumented migration rather than formal employment. Most Thai workers in palm plantations originate from border communities in Satun, Songkhla, Narathiwat, and Yala provinces and enter Malaysia through informal cross-border movement, primarily facilitated by social employment through personal networks of relatives, friends, or community members already employed in Malaysian palm plantations.

This chain-like migration dynamic means that initial migration decisions are rarely mediated by authorized employment agencies; instead, individuals already employed in the palm plantations—typically family members or close acquaintances—serve as the primary employment channel, providing information, arranging transportation, and, in some cases, even accompanying new migrants across the border. In some cases, informal border labor contractors (*Mr. Nai* or *Mr. Kalo*) coordinate border-crossing and employment arrangements. A small fee, typically between 1,000 and 3,000 Baht (approximately 120-360 MYR), is charged, which is significantly lower than broker fees in the Indonesian or Bangladeshi recruitment context and does not create a significant debt burden.

The border crossing takes advantage of the open nature of the Thai-Malaysian border: workers generally cross via informal routes, jungle trails, or secondary border crossings, rather than official immigration checkpoints, entering Malaysia without visas or work permits. This method of entry results in Thai agricultural workers becoming undocumented migrants, outside the protection of the formal bilateral labor framework regulating Thai-Malaysian labor migration. This informal and network-driven recruitment system in Thailand differs structurally from both Indonesia’s system, which uses recruitment agencies, and Bangladesh’s government-to-government system, but shares a crucial institutional outcome: workers without legal immigration status and the protection dependent on that status.

#### 6.2.2 Legal status, documentation, and employer control

The lack of documentation creates fundamental institutional imbalances in vulnerability among the three groups. The majority of undocumented Indonesian workers are at high risk of exploitation, as employers confiscate their passports, their only identification document, effectively hindering their movement. Similarly, Bangladeshi workers, even those entering through formal government-to-government channels, face a similar institutional conflict: their work permits are held by their employers, mirroring the dependence structure of undocumented workers within the formal system. These arrangements limit their ability to change employers or access grievance mechanisms without risking permit revocation and deportation, while debt burdens hinder voluntary departure.

In practice, the documented status of Thai workers reflects more closely the status of undocumented Indonesian workers than the officially protected status stipulated in bilateral agreements. Because the main entry routes are informal and undocumented, most Thai plantation workers in border areas lack valid work permits and are in Malaysia without authorization. This undocumented status denies them access to all aspects of formal labor protection, including minimum wage enforcement, occupational safety standards, social security, and access to labor and labor relations courts, creating a complete institutional protection vacuum.

Employers are aware of the workers’ undocumented status and exploit it structurally, such as by confiscating passports or identification documents. While not as widespread among Thai workers as among Indonesian workers (partly because some Thai workers do not carry passports for border crossings but use border passes or cross without documentation), it still occurs. More importantly, the ongoing threat of immigration enforcement and deportation serves as an effective punitive mechanism, deterring workers from filing wage disputes, reporting safety violations, or contacting external aid organizations. Unlike legally documented migrant workers, who can, in principle, appeal employers’ work permit obligations, undocumented Thai workers have no bargaining power in enforcing contractual terms.

The Royal Thai Embassy and Consulates in Penang and Kota Bharu provide limited consular assistance, but consular protection is virtually impossible for remote plantation workers who cannot travel freely and fear that contacting the consulate might expose their undocumented status to Malaysian immigration authorities. The reality of this institutional protection vacuum and the strategic threat of deportation are detailed in the following narrative by an undocumented Thai worker:

*“Coming to work here is really cheap because we just use informal networks, meaning we do not arrive with the massive debt bondage that the Bangladeshi guys have to face. But honestly, because we do not have proper papers, we are left in a complete legal vacuum with zero protection. If we try to complain about minimum wage violations or report that our safety equipment is broken or missing, the managers just use our legal status against us, threatening to call immigration right away. We constantly have to be on high alert—whenever there’s a labor inspection, we have to run and hide deep in the fields so we do not get arrested under the Immigration Act. This constant fear of getting caught completely strips away our power to bargain, leaving us relying entirely on the employer’s mercy just to make it through each day.”*— (Transcript Identifier: TH-INT-14, Undocumented Male Harvester, Kedah Border Zone).

#### 6.2.3 Labor standard enforcement

Malaysia’s Employment Act 1955, together with the Minimum Wages Order 2024, sets a minimum wage (1,700 ringgit effective February 2025), limits working hours, establishes fair contract principles, and outlines formal dispute resolution mechanisms. In practice, law enforcement capacity remains severely lacking across all ethnic groups in the dryland plantation environment. Workers frequently report passport confiscation under the pretext of “security,” aligning with ILO forced labor indicators (International Labour Organization, 2012). Section 26A of the Trade Unions Act 1959 prohibits union membership. Formal complaint mechanisms are difficult to access due to language barriers, employer retaliation risks, geographic remoteness, and low legal awareness, compounded by infrequent labor inspections despite comprehensive regulations.

Bangladeshi workers face the most severe obstacles to accessing labor protection systems, with limited Malay or English language skills hindering their ability to contact complaint systems. This combination of structural isolation and document withholding is described by a Bangladeshi laborer:

*“The management took my passport away the second I arrived at the estate, claiming it was for ‘safekeeping,’ which completely stripped away my freedom to go anywhere. Because I do not speak a word of Malay or English, I have absolutely no idea how to deal with the government laws or contact the Department of Labour to report how they are cutting our wages. We’re basically trapped in a closed loop—our lack of language skills completely blocks us from getting help from the very systems that are supposed to protect us.”*— (Transcript Identifier: BGD-INT-07, Male General Worker, Kedah).

Undocumented Indonesian workers cannot access official protection without risking deportation. The status of Thai workers, upon closer examination, is even more vulnerable than previous research has indicated, as the majority are undocumented migrants. Thai workers in agricultural fields face similar exclusion from official labor protection mechanisms as undocumented Indonesian workers; they cannot file wage complaints with the Department of Labor, cannot utilize minimum wage laws, cannot access worker compensation programs, and cannot report employers for safety violations without risking arrest and deportation under the Immigration Act of 1959/1963.

Ironically, the Malay language skills of Thai workers, which could theoretically facilitate access to complaints, are impaired by their undocumented status, making their use of language a burden rather than a benefit. Geographical proximity to Thailand, which allows for informal entry of Thai workers, also serves as their primary de facto protection mechanism: the ability to temporarily return to Thailand in case of severe exploitation or repression by authorities. Or in the case of workplace injuries, the option to leave the country is more of a substitute than a complement to legal labor protections and reflects a complete institutional gap affecting undocumented Thai workers. Labor inspections in border agricultural areas provide virtually no protection for undocumented Thai workers. The routine failure of state enforcement and the complex role of language are detailed in the following account by an undocumented Thai worker:

*“Even though many of us can speak Patani Malay and can talk to the local officials, being undocumented just makes this skill totally useless. If we try to bargain or speak up about not getting the minimum wage, the managers simply threaten to report us to immigration. Plus, the labor inspections around here do not actually protect us at all. Whenever an inspection is coming up, the boss gets a heads-up or an informal warning from their local contacts. The manager then orders all of us undocumented Thai workers to go hide deep inside the plantation plots or cross back into Thailand for the day. This completely cuts off our daily piece-rate income and leaves us relying entirely on the employer’s mercy just to survive.”*— (Transcript Identifier: TH-INT-12, Undocumented Male Harvester, Kedah Border Zone).

### 6.3 Social dimension: migration networks, social capital, and community integration

Social networks represent the third analytical dimension, defining recruitment pathways, the distribution of social capital and information, end-user support systems, and the level of community integration that impacts worker wellbeing. The three ethnic groups exhibit distinctly different network structures, leading to diverse social outcomes within the palm oil plantation context.

#### 6.3.1 Migration networks and social capital

Indonesian Workers: Indonesian workers benefit from dense migration networks that have accumulated over decades through the continuous movement of labor to Malaysia. This chain migration model, in which existing workers facilitate and inform relatives and community members about migration, mitigates information asymmetries and migration costs for documented immigrants. These networks facilitate access to a wider range of employment opportunities and provide informal support upon arrival. However, the infrastructure of these same networks is exploited by *pengguru* entrepreneurs who take advantage of trusted relationships to facilitate illegal immigration and create debt dependence. The dual nature of Indonesian networks—both protective and exploitative—reflects the entrenching of informal migration intermediaries within existing social structures. This dual reality of community support intertwined with informal exploitation is described by an Indonesian worker:

*“Our community network inside the estate is actually really strong. We’ve set up our own ways to help each other out and share communal spaces just to get through the isolation together. But honestly, this same network is a double-edged sword. When my relatives wanted to come join me, we relied on a panggurus from our very own village—we trusted him completely just because we are from the same place. Instead of a safe trip, he jacked up the border-crossing fees to over 1,200 MYR and trapped them in a high-interest debt deal. The trust we had in our own people was basically weaponized against us.”*— (Transcript Identifier: IDN-INT-08, Male Harvester, Kedah).

Bangladeshi Workers: Bangladeshi workers enter Malaysian plantations with social networks within a relatively underdeveloped agricultural context. Most recruitment in Bangladesh is carried out through formal and semi-formal brokers (*dalal*) rather than through existing personal networks among migrant workers. This leads to an immediate dependence on intermediaries rather than on a social community upon arrival. This structural isolation is exacerbated by language barriers—Bangladeshi workers have limited proficiency in Malay or English, which limits social integration beyond their national peers. The lack of strong community networks leaves Bangladeshi workers disproportionately dependent on employers for information and services, further reinforcing structural power imbalances. The psychological toll of this systemic isolation is captured in the following narrative:

*“Life out here on the plantation is just so isolating because we do not have any kind of community network to look out for us. We did not get here through family connections; we were processed by commercial brokers (dalal) who basically abandoned us the second we reached the estate. Because the language barrier is so bad, I cannot communicate with Malaysian society, local officials, or even the Indonesian workers at all. Our whole social world is pretty much limited to the few Bangladeshi guys in our barracks, which leaves us completely dependent on the manager for basic necessities and information.”*— (Transcript Identifier: BGD-FGD-02, Male General Worker, Perlis).

Thai Workers: The social network of Thai workers has a distinct transnational character, shaped by their proximity to their country of origin. Instead of relying on a localized Thai community based in Malaysia, Thai workers in border plantations maintain intensive transnational social ties to communities in Satun, Songkhla, and Narathiwat provinces, facilitated by short travel distances (often under 2 h), mobile telephone connectivity, and relatively porous border arrangements for short-term movement. This cross-border network structure provides meaningful social and psychological support—workers can stay in regular contact with their families and participate in cross-border community activities.

Access to social institutions in Thailand serves as a vital emotional buffer, but it does not translate into a strong domestic support structure like the long-established Indonesian community network in Sabah. The lower density of Thai workers in any single farming area has hindered the establishment of destination-based ethnic institutions, such as religious sites, specialized shops, or formal mutual aid groups. Consequently, their support remains externalized across the border. The unique, proximity-based coping mechanism of this cohort is detailed by a Thai border worker:

*“I can talk to my family every day on my phone without worrying about expensive costs, and I can physically be back in my home province in just a few hours if there’s an emergency or if things at work get too bad to handle. Just knowing that I have an easy way out really helps with the mental pressure of plantation life. It honestly makes up for the fact that we do not have any formal Thai community centers inside these Malaysian estates.”*— (Transcript Identifier: TH-FGD-03, Male Harvester, Kedah Border Zone).

#### 6.3.2 Housing conditions and geographic segregation

The accommodation provided by employers constitutes physical control over all laborers, isolating them from urban services, alternative employment opportunities, legal assistance, and support networks. Common living conditions across all groups include substandard structures and inadequate sanitation, crowded dormitories, contaminated or insufficient water supply, limited or nonexistent healthcare facilities, and remote locations that make independent travel difficult without employer-controlled transportation.

Under these generally unfavorable conditions, differences by nationality can be observed. Indonesian workers who have worked long terms and have families are sometimes provided with slightly better accommodation, while undocumented Indonesian workers tend to live in the worst quality structures and have the least chance of seeking outside help. This layer of spatial neglect and legal vulnerability is described by an undocumented Indonesian laborer:

*“The housing they give to undocumented workers like us is completely hidden away in the most remote parts of the estate. It’s basically just dilapidated wooden barracks with no reliable clean water or electricity at all. Because we do not have papers, we are forced to just accept these dirty, crowded conditions—we cannot even ask them to fix anything. The management purposely uses this remote location to cut off our mobility, making it absolutely impossible for us to go out and look for help or legal aid unless we use the company’s own transport.”*— (Transcript Identifier: IDN-INT-10, Undocumented Male Harvester, Kedah).

Bangladeshi workers report the most severe congestion, including cases of temporary structures not designed for residence. The housing conditions of Thai workers are still very rarely recorded in the academic literature. Field evidence from the Thai-Malaysian border area suggests that conditions are generally comparable to those of Indonesian workers in equal roles. Although the smaller density of Thai workers in each farm may reduce the most severe congestion recorded in Bangladeshi hostels, the proximity of Thai workers to the border and, in some cases, to Thai border communities, makes it possible to leave unofficially—that is, the possibility of returning to Thailand temporarily—which serves as a partial counterweight to the geographical isolation that is absent in the Indonesian or Bangladeshi workforces.

#### 6.3.3 Social integration and exclusion

Indonesian migrant workers benefit greatly from their cultural and linguistic proximity to Malaysian society. Shared Austronesian linguistic roots, similar Islamic practices, and decades-long community establishment in Sabah create conditions for significantly superior social integration compared to other groups. The established infrastructure of Indonesian communities (ethnic businesses, mosques, informal networks) provides both material and spiritual support, mitigating the effects of social exclusion in its most severe forms.

Bangladeshi workers face the most severe social barriers arising from the convergence of linguistic and cultural differences, physical isolation in remote areas, and discriminatory practices documented in the Malaysian social context. The social life of most Bangladeshi workers is limited to their Bangladeshi colleagues. With very little interaction with Malaysian society, Indonesian workers, or Thai workers, this isolation increases the mental burden of debt, family separation, and rights violations. The profound structural alienation experienced by this cohort is illustrated in the following narrative:

*“Our whole lives are basically trapped between the barracks and the fields every single day. Since we do not speak a word of Malay and do not share any culture with the locals, we face a lot of racism and social exclusion out here. We’re completely cut off from Malaysian society, and we are even isolated from the Indonesian and Thai workers on the same estate. Having absolutely no community support here just makes everything so much worse—it heavily adds to the mental stress of carrying huge recruitment debts and being separated from our families for so long.”-* (Transcript Identifier: BGD-FGD-03, Male General Worker, Perlis).

The social integration of Thai workers in Malaysia is influenced by several specific factors. In border communities with a predominantly Thai culture, particularly in areas with predominantly Thai-Malay Muslim communities, Thai workers are more socially accepted than Bangladeshi workers. This is a result of physical proximity, cultural familiarity between border populations, and, in some cases, shared religious heritage with the Thai-Muslim communities in Malaysia.

However, most Thai workers are employed in plantations where Thai culture is less prevalent, and the number of Thai workers in each plantation is small, making community formation difficult. Therefore, the social experience of Thai workers in Malaysia is characterized by strong transnational ties to their home communities rather than robust intra-national integration. This cross-border connectivity helps maintain wellbeing and identity, but receives limited institutional support within the destination country’s migration authorities.

### 6.4 Individual dimension: human capital, debt obligations, health outcomes, and psychological wellbeing

The personal dimension considers how the individual characteristics and personal resources of the workforce interact with social structures, institutions, and conditions in four areas: human capital, debt, occupational health, and mental wellbeing. The overall patterns of the groups are consistent across all four areas, with Bangladeshi workers experiencing the most severe vulnerability, followed by Indonesian workers, and relatively higher levels of agency among Thai workers. This ranking is presented only once and summarized in Table 2. Subsequent subsections report only the specific empirical evidence, and the interpretation of the mechanisms that led to the ranking will be discussed in Section 7.

**Table 2:** Cross-dimensional cumulative vulnerability profiles by nationality group.

Dimension	Thai workers	Indonesian workers	Bangladeshi workers
Structural	Low push factor; border geographic concentration; moderate exit capacity; wage comparable to Indonesian workers	Historical dominance; declining share; diverse task assignment; wage below minimum	High push factor; highest-risk task concentration; wage lowest; highest wage theft
Institutional	Stronger bilateral framework; mostly formal channels; better documentation custody; basic Malay competency aids access	Dual formal/informal channels; most undocumented; passport confiscation prevalent	G2G subverted; employer controls permits; fee inflation extreme; most severe enforcement barriers
Social	Strong transnational ties to Thailand; limited in-country community; border cultural familiarity; informal exit option	Dense established networks in Sabah; cultural-linguistic proximity to Malaysia; dual protective/exploitative network character	Underdeveloped networks; broker dependency; severe linguistic/cultural isolation; most acute social exclusion
Individual	Lower debt (≈ MYR 120–360/THB 1,000–3,000); Malay language capital; domestic labor market exit option; psychological buffer via transnational ties	Moderate debt; migration-specific human capital among experienced workers; chronic legal stress (undocumented)	Extreme debt (USD 4,400–6,600); >70% wages to debt; highest health risk; most severe psychological distress

#### 6.4.1 Human capital and pre-migration resources

The level of education, language ability, technical skills, and prior migration experience of the workers contributed to their ability to work on the plantations and access various services. Indonesian participants with prior work experience in Malaysia utilized their accumulated knowledge of the labor market, legal system, and supporting organizations, while first-time migrants in this group lacked such knowledge.

Bangladeshi participants often arrived with very little specific preparation for Malaysia, reporting minimal pre-departure orientation from employment agencies. This lack of institutional preparation left them structurally unequipped to comprehend their contract terms or navigate the localized estate environment upon arrival. This deficit in pre-migration human capital is described by a Bangladeshi laborer:

*“They just shipped us off to the plantation without any orientation or real information about what oil palm harvesting is actually like. The recruitment agency back in Dhaka only cared about collecting their fees, leaving us completely in the dark about the language, the laws, or our basic rights in Malaysia. When we got here, because we were so completely unprepared, we had to rely entirely on whatever the boss said about our duties and piece-rates. It completely stripped away our power to even realize we were being exploited, let alone fight back.”*— (Transcript Identifier: BGD-INT-09, Male General Worker, Perlis).

Thai participants reflected Thailand’s relatively high secondary education enrollment rate, and many possessed basic Malay language skills acquired through the linguistic continuity of the border region, where Patani Malay is widely spoken. Participants reported using this capability to understand instructions and negotiate terms with plantation managers. Proximity to Thailand also allowed Thai workers continuous access to labor market information in their home country. The strategic deployment of this localized linguistic human capital as a defensive resource is detailed in the following account by a Thai border worker:

*“Since we grew up in Satun, right on the border, most of us speak Patani Malay fluently. It’s basically the same language the estate managers use out here, and that gives us a huge edge over workers who come from far away. We do not need any translators to understand what we are supposed to do. But more importantly, we can actually negotiate our piecework rates and stand up for ourselves whenever a manager tries to cheat us or make unfair deductions. Plus, we are always checking our phones for labor market updates back in Thailand. When you combine that with our language skills, it really gives us the power to look out for ourselves.”* (Transcript Identifier: TH-INT-15, Male Harvester, Perlis Border Zone).

#### 6.4.2 Debt obligations and financial vulnerability

Workplace debt and financial dependency vary considerably depending on nationality and recruitment channels. Bangladeshi participants reported the highest recruitment expenses, totaling between US$4,400 and US$6,600. While their monthly wages under the performance-based system were generally below 900 Malaysian Ringgit (MYR), over 70% of these workers allocated at least half of their monthly income to debt repayment, often servicing multiple debts concurrently with interest rates as high as 30%. Consequently, most reported an inability to remit payments or accumulate savings. Wage fraud was also most frequently reported among this group, widening the gap between expected and actual debt repayment ability. This severe financial entrapment, illustrated in the recruitment testimony presented in Section 6.2.1, leaves Bangladeshi workers with virtually no capacity to exit or contest their conditions.

Indonesian participants entering through unofficial channels paid border-crossing fees of approximately 1,000 MYR or more. Workers who entered through official recruitment channels reported lower entry costs, though additional unauthorized fees were occasionally charged by local agencies. Longer average employment periods and stronger social networks within the Indonesian cohort were associated with more gradual debt repayment over time.

Conversely, Thai participants reported the lowest entry and recruitment costs among the three groups, ranging from approximately 1,000 to 3,000 Baht (equivalent to roughly 120 to 360 MYR). These minimal upfront expenses were typically liquidated through plantation wages within two to 3 months of arrival. The high degree of agency resulting from this low-debt profile is detailed in the following narrative by a Thai border worker:

*“Getting over here barely costs us anything because we lean on family and village connections—you do not need a lot of money upfront. It cost me less than 2,000 Baht to cross the border from Satun, and I just used my family’s savings instead of borrowing from loan sharks. Since I made that money back in just the first two months of working, I do not feel trapped or dependent on the boss at all. If the managers try to make unfair deductions or cut our piece-rates, we can just say ‘enough is enough,’ pack our bags, and go back to farm our own land across the border.”*— (Transcript Identifier: TH-FGD-02, Male Harvester, Kedah Border Zone).

Following debt liquidation, many Thai workers reported exercising their mobility by leaving plantation work and later returning when economic conditions favored them. Furthermore, Thai participants frequently utilized proximity-based informal channels at border checkpoints to remit their earnings back to Thailand.

#### 6.4.3 Occupational health and safety

Palm oil plantation work exposes all worker groups to pesticides and fertilizers, resulting in physical injuries from harvesting equipment and falling palm fruit, heat stress, dehydration, and insect-borne diseases such as malaria and dengue fever. Participants from all nationalities reported inadequate personal protective equipment, insufficient workplace health care despite legal requirements, and unwillingness to take sick leave or report injuries due to piecework wages directly converting absenteeism into lost income. Bangladeshi participants, primarily employed in handling, transporting, and high-risk roles, reported the highest rates of accidents and disease exposure.

Women in all groups involved in pesticide spraying reported direct skin and respiratory exposure to chemicals with inadequate protective equipment. This gendered occupational hazard is underscored by a female worker who described the severe health compromises forced by the piecework structure:

*“We are routinely assigned to handle highly toxic herbicides and chemical fertilizers without the provision of standard personal protective equipment (PPE), such as industrial masks or heavy-duty gloves. Many of us suffer from chronic skin rashes, chemical burns, and respiratory distress. However, because our earnings are strictly tied to a piece-rate system, taking a sick day immediately results in a total loss of daily income. We are effectively forced to jeopardize our long-term health simply to secure our daily survival.”* — (Transcript Identifier: IDN-FGD-04, Female Maintenance Worker, Kedah).

Systematic occupational health data on Thai workers remain scarce. Field evidence points to similar patterns of equipment shortages, heat stress, and working while sick or injured, including two group-specific practices: short-term return to Thailand for medical treatment and prior familiarity with the physical demands of palm oil work from Thailand’s domestic industry. The distinct cross-border healthcare strategy utilized by Thai workers as an institutional safety valve is detailed in the following narrative:

*“When we experience workplace injuries from falling fruit or contract illnesses like dengue fever, the estate management rarely provides adequate medical assistance or insurance coverage. Because we are near the border, our primary survival strategy is to temporarily return to Thailand to access treatment under our domestic universal healthcare system. It is far safer and more affordable to cross back into Satun or Songkhla for proper hospitalization, recover at home with family support, and then return to the plantation once we are physically fit to resume manual labor.”* — (Transcript Identifier: TH-INT-09, Male Harvester, Kedah Border Zone).

#### 6.4.4 Psychological wellbeing

Participants in all three groups described psychological distress linked to debt and financial insecurity, separation from family, fear of deportation among undocumented individuals, exploitation at work, and uncertainty about the future. Bangladeshi participants described the most severe distress, citing a combination of significant debt, linguistic and social isolation, and, among those traveling by sea, traumatic experiences including human trafficking, maritime violence, and witnessing the deaths of other migrants. Undocumented Indonesian participants described chronic stress linked to legal insecurity and the threat of deportation, making them hesitant to seek mental health services or social support. This pervasive fear of state enforcement is illustrated by an undocumented Indonesian laborer who stated:

*“We live in a constant state of hyper-vigilance. Every unfamiliar vehicle entering the estate grid triggers an immediate panic response because we assume it is an immigration raid. This chronic anxiety forces us to avoid seeking medical attention at local clinics even when severely injured or ill, as the risk of arrest and subsequent deportation outweighs our immediate health concerns.”* > — (Transcript Identifier: IDN-INT-11, Undocumented Male Harvester, Kedah).

Thai participants indicated that regular cross-border contact with family, convenient due to short travel distances and low communication costs, and the option to return home, was their primary source of psychological relief.

### 6.5 Cross-level synthesis: cumulative effects and common structural barriers

A four-dimensional framework reveals that the working conditions of Thai, Indonesian, and Bangladeshi migrant workers in Malaysian oil palm plantations result from the interaction among macroeconomic structural forces, institutional frameworks, social network structures, and individual resources and vulnerabilities, rather than any single factor. Macroeconomic structural forces in the global oil palm supply chain create a demand for cheap, controllable labor in secondary oil palm plantation labor markets. Institutional frameworks dictate which nationalities have access to which recruitment channels and protections. Social networks serve as intermediaries in managing costs, channels, and social capital upon arrival. Individual factors determine the extent to which workers can negotiate, resist, or escape exploitation.

Three common barriers affect all ethnic groups regardless of whether Thai workers have structural advantages or network advantages compared to Indonesian workers: (1) the employer-controlled work permit system, which binds residency rights to a single employer and hinders collective bargaining through a ban on unions; (2) geographical isolation in remote palm-growing areas, which impedes access to legal aid, healthcare, and grievance mechanisms for all groups; and (3) the performance-based wage system, which creates income volatility and incentivizes all ethnic groups to work despite poor health, regardless of health status. These common barriers reflect the systemic nature of exploitation in Malaysia’s palm oil industry and indicate that policy interventions targeted at individual ethnic groups, while necessary, must be supplemented by structural reforms that address the institutional architecture of labor regulation in palm plantations.

## 7 Discussion

This research analyses the working conditions, wages, and labor rights of Thai, Indonesian, and Bangladeshi migrant workers in Malaysian oil palm plantations using a four-dimensional conceptual framework that integrates structural, institutional, social, and individual factors. A key finding is that ethnically differentiated vulnerabilities are not accidental or culturally determined, but rather arise systemically through the interaction of global commodity chain pressures, governance failures, network structures, and individual limitations. The following discussion not only confirms existing theories but, in each case, uses a comparative analysis of three data sources to identify points where the conceptual framework imposes explanatory limitations and to determine the mechanisms necessary to extend the framework beyond those limitations. Collectively, these extensions converge on a single integrated model of how similar structural positions are systematically transformed into life inequalities.

### 7.1 Confirmation and extension of the labor market segmentation theory

Piore's (1979) dual labor market theory posits that developed economies create a structural demand for secondary labor—low-wage, unstable, difficult-to-move, and dangerous jobs that domestic workers avoid, replaced by migrant workers whose weak bargaining power forces them to accept such conditions. Half a century later, this central assumption of the theory is strongly confirmed here: all three groups belong to this secondary labor pool, characterized by piecework wages below the minimum wage of 1,700 MYR, chronic instability, and physical risks. However, the confirmation is not the crucial point. The crucial point lies in the anomaly revealed by a comparison of the three origins: the secondary labor pool is not internally homogeneous as the segmentation theory assumes, but rather hierarchically arranged. This hierarchical structure is called stratification within the secondary market: within a single secondary market, workers are ranked according to nationality through a combination of the political economy of their country of origin, bilateral regulatory design, and employer job allocation. Bangladeshi workers are at the bottom (highest risk jobs, lowest wages, highest wage fraud), Indonesian workers are in the middle, and so on, with Thai workers in a more advantageous position—yet all three groups clearly remain within the secondary market. Market segmentation theory, which is designed to describe the boundary between primary and secondary markets, does not address stratification within secondary markets. Therefore, it cannot explain why workers in the same positions have vastly different levels of vulnerability.

This hierarchical mechanism is what we call structural ability to leave: the degree to which macroeconomic structural conditions—geographic proximity to origin, availability of choices in the domestic labor market, debt burden, and movable social capital—allow workers to transform formal options for resignation into practically feasible options. Ability to leave varies analytically both from labor market position (worker status) and from human capital (what each worker possesses). It relates to the structural possibility of leaving work. The distribution of this ability is systemic: Bangladeshi workers have almost none (debt of US$4,400–US$6,600 makes leaving work economically catastrophic), Indonesian workers have some ability (networks provide alternative routes, but their undocumented status hinders legal mobility), and Thai workers have high ability (proximity, choices in the domestic palm oil sector, and low debt make continuing work in palm oil plantations voluntary). Theoretically, structural ability to leave bridges the gap between status and vulnerability; that is, this research identifies how similar structural statuses are transformed into different levels of life insecurity. The scope of this research is not limited to this case alone. Wherever secondary labor markets draw workers from diverse countries of origin, varying in distance, debt risk, and domestic options—a common condition in agro- and low-wage economies across Southeast Asia and elsewhere—the structural ability to leave should be able to predict the order of vulnerability within, transforming descriptive observations (some migrants are worse off than others) into generally applicable causal conclusions.

### 7.2 From distorted to absent infrastructure: extending the precarious-infrastructure framework

Sunam (2022)‘s conceptual framework on unstable infrastructure highlights that the migration management system—comprising recruitment mechanisms, employer-linked work permits, residency conditions, and employer-controlled housing—fails not only to protect migrants but also actively creates and exacerbates risks. From this perspective, instability arises from the design and distortion of existing institutions. Our evidence strongly corroborates this conceptual framework, and in the case of Thailand, it further identifies structural dimensions the framework was not designed to explain.

The case of Bangladesh is the clearest example that proves the validity of this conceptual framework. The intergovernmental channels designed as protective mechanisms were instead dominated by private intermediaries charging fees far exceeding the official caps, leading to pre-employment debt that binds workers to employers before they even begin working a single day. The work permits held by employers create the same dependency for documented workers, while the geographical isolation of industrial zone housing—coupled with employer-controlled transportation and communication—expands instability from recruitment and daily life to the very mechanisms of grievance redress. This is the instability arising from distortion in the sense Sunam (2022) describes, meaning protective institutions exist but are distorted, resulting in documented workers receiving less effective protection than officially stipulated.

The case of Indonesia only partially confirms this conceptual framework, and in doing so is also a correction of this conceptual framework. The coexistence of formal and informal channels creates two levels of unstable infrastructure: legally documented workers face employer-bound permits and limited mobility, while undocumented workers face complete legal barriers. However, Indonesia’s dense network in Sabah (Ford, 2006) has helped reduce this insecurity in part through information solidarity. And the informal suspension of disputes, which is a protective dynamic that Sunam’s emphasis on risk creation overlooks, so this conceptual framework needs to recognize mitigation from the network as a counterforce, not just an expansion of risk.

The case of Thailand demonstrates the absolute necessity of elaboration. The precariousness of Thai labor is not caused by distorted institutions but by complete exclusion from them. Unauthorized entry through family networks and communities means no work permit, no bilateral obligations, no responsible employer, and no official records. This is not a flawed protective framework but a vacuum of protection. We refer to this mechanism as the absence of infrastructure: instability arises from exclusion from regulatory infrastructure rather than distortion of that structure. The theoretical significance lies in two aspects. First, it reveals that Sunam’s conceptual framework is, in fact, a theory of the existence of hazardous institutions, while instability can also stem from their absence. Second, absence possesses a distinctive quality that distortion lacks: invisibility. When there is no contract, record, or responsible employer, this form of instability remains undetectable by inspection tools (audits, certifications, complaint systems) designed to identify distortion.

Distortion and absence should be understood as two poles of institutional continuity, with Indonesia’s dual channel case lying midway between these two poles. This leads to the unified conclusion that instability results from the existence and direction of institutions, which may be absent, exist and provide protection, or exist but are distorted. The boundary conditions are broad—whether informal or undocumented migration routes parallel formal migration routes or not—and the impact on tracking is direct, which will be discussed in Section 7.6.

### 7.3 The politics of Unfreedom across three origin trajectories

Li (2017a,b) political concept of unfreedom states that the agricultural labor system combines both freedom and unfreedom. The workers are granted nominal rights of choice, while structural conditions block meaningful choices, resulting in obedient labor while maintaining an image of compliance. Our comparison provides systematic empirical data, though not further developed, on the model Li described: that the definition of freedom/unfreedom is source-specific, with three distinct definitions.

For Bangladeshi workers, the lack of freedom appears in its most complete form, which we call forced consent. At the official level, the consent is real—the workers sign, pay US$4,400–6,600 in employment fees, and emigrate, but the consent is built on previous structural conditions: Non-immigration is financially impossible, and non-compliance with the employer’s demands will bring with it severe economic consequences through debt. Therefore, the category of voluntary workers is emptied, while the legal form remains. The mechanism of work is debt, and it works in the future—binding labor before employment begins.

For Indonesian workers, the lack of freedom stems more from their legal status than from debt. Undocumented workers retain the legal freedom to travel abroad and are not bound by debt to the same degree as workers in Bangladesh, but the risk of deportation makes any complaint invalid. The punitive mechanism is therefore preventative; that is, it is not implemented through the actual exercise of state power, but rather through anticipation, which workers accept as a reason not to pursue their rights. This clarifies Li′s explanation of state power, stating that within this structure, power is most effective when implemented preventatively.

For undocumented Thai workers, this case especially strains Li’s framework. Thai workers have real freedom, not just nominal freedom to leave their jobs: proximity, low debt, and local agricultural alternatives mean that leaving their jobs in the plantation does not lead to financial failure. Li’s logic predicted that workers with this much structural freedom should have been given better conditions or stronger bargaining power, but they did not get it back. Thai workers continue to accept wages below the minimum wage, dangerous working conditions, and a complete lack of labor protection. And when they express dissatisfaction, they do so by traveling back to Thailand rather than negotiating at work. This disorder forces the ideological differences that frame existing ideas to fail: freedom to leave (the ability to leave an employment relationship) is unlike freedom of expression (the ability to negotiate terms within that relationship). Thai workers are in the first place and lacking in the second, because the informal and undocumented nature of employment leaves them without any institutional channels to delay.

Separating these two freedoms is the actual development of Li′s concept, which has an impact on analysis and policy. The two freedoms have different structural determinants-freedom to go abroad depends on proximity, debt and other choices, while freedom of opinion and expression depends on recorded institutional channels-so they respond to different and irreplaceable interventions, which is the problem expounded in Section 7.6 The combination of these two freedoms in previous studies obviously covered up the blank shown by the case of Thailand.

### 7.4 Toward an integrated cross-level model

The three extensions above are not independent improvements to separate theories, but rather aspects of a single integrated model that the four-dimensional framework is designed to describe. Integration logic is sequential and interactive: structural positions define the boundaries of possibilities; institutions translate those positions into protection or exposure; social networks mediate this translation; and individual actions are carried out within the established boundaries. Three cross-level dynamics describe how the levels interact.

Dynamic 1—The link between structure and institutions is nonlinear, with variable signs. Institutions do not merely add layers of protection or risk to structural positions; they act as multipliers with signs that can reverse structural forecasts. Institutional exclusion can negate strong structural positions—Thai workers have low drive factors and high exit potential, coupled with a protection vacuum, creating vulnerabilities that their structural advantages cannot compensate for—while institutional channels can maximize weak structural positions—Bangladesh’s dominating G2G system transforms this disadvantage into near-total dependence. This is why one-dimensional models that only consider structure misrepresent the case of Thailand, as they fail to represent institutions that negate structural advantages.

Dynamic 2—Networks serve as intermediaries to mitigate structural constraints but remain constrained by institutions. Indonesia’s robust network in Sabah provides genuine and measurable support yet fails to facilitate access to formal complaint processes, minimum wage enforcement, or social security. Thus, network capital is asymmetrically constrained: it can reduce workers’ risks within institutionally defined systems but cannot substitute for institutional engagement. This both affirms and extends Massey et al. (1993)‘s cumulative causation framework by identifying the upper limits of what networks can achieve. The Thai case offers a lesson: a sparse domestic network is compensated by cross-border psychological ties with Thailand, yet these also lack institutional voice—confirming that the upper limit originates from institutions, not network density.

Dynamic 3—Individual actions occur within defined structural and institutional frameworks. The bilateral separation between action and structure should be replaced by the image of a corridor: structures and institutions define the breadth of the corridor in which choices are meaningful. Individual potential (language, education, previous migration experience) determines where a worker will work within that corridor, but the corridor cannot be widened from the bottom. The Bangladesh corridor is very narrow—compliance is mandatory or risk deportation due to unpaid debts. The Thai corridor is much wider—debts are manageable, resignations are possible, and Malay language proficiency provides an informational advantage—but even the wider corridor does not exclude a powerful voice in the workplace.

When combined, these three dynamics give rise to the model’s central proposition: that differential vulnerability by nationality is the joint product of structural position, institutional translation in terms of symbols and scale, and institutional ceilings of networks. Workers perceive this as the boundary of actionable agency. No single framework—be it segmentation, infrastructure, or lack of autonomy—can generate this proposition on its own. It emerges only through the intersection of these four-dimensional frameworks.

### 7.5 Theoretical contributions of this study

In summary, this research has created five interconnected paradigms for the literature on plantation labor and migration in Southeast Asia. First, the stratification within the tertiary level. The tertiary agricultural labor market is not homogeneous, but is internally organized by nationality, through the interaction of the political economy of the country of origin. Sectoral regulation and employer assignment. With reference to Piore (1979), this study identifies the mechanism—classification within qualification levels—that causes some different vulnerabilities among workers in the same position, and offers a sharper analytical tool than indiscriminate grouping for agricultural environments of diverse origins.

Secondly, the ability to escape structurally unfair employment conditions is a dimension of labor market status. It is defined as the degree to which macroeconomic structural conditions (proximity, domestic choice, debt, social capital) enable workers to truly escape unfair employment conditions, not merely formally. This concept differs systemically across different originating populations and mediates a shift in structural status to genuine vulnerability that neither stratification theory nor human capital theory can explain. This concept is applicable to farm labor markets and low-wage agricultural labor markets in general.

Thirdly, the lack of infrastructure is a distinct mechanism of insecurity. Expanding on Sunam (2022), the complete exclusion of undocumented migrant workers from institutional infrastructure is a mechanism of insecurity that operates differently from institutional distortions and, more importantly, creates invisible vulnerabilities—exploitations that go undetected by traditional labor monitoring systems. The case of undocumented Thai migrant workers provides empirical evidence with direct implications for monitoring informal labor across the region.

Fourth, freedom to leave employment versus freedom of expression as separate dimensions of power in the labor market. Revised research by Li (2017a,b) found that freedom to leave employment relationships and freedom to negotiate terms within those relationships can be structurally separated—the Thai case demonstrates freedom to leave employment without freedom to negotiate terms—and these two freedoms have different determinants and require different policy mechanisms and are not interchangeable.

Fifth, the four-dimensional framework as a cross-level analysis. Methodologically, the integration of structural, institutional, social, and individual levels reveals three cross-level dynamics—nonlinear structural-institutional linkages, networks limited by institutional boundaries, and power bandwidth determined by structures—which no single framework can fully encompass. Therefore, it provides a reproducible comparative approach for multi-source labor settings beyond the current case.

### 7.6 Implications for policy and practice

The theoretical work aforementioned has direct implications for policy formulation, transcending specific empirical contexts. Firstly, findings on secondary internal stratification and the ability to exit structural systems demonstrate that policy interventions targeted at nationality-specific vulnerabilities—bilateral agreements, consular services, pre-travel orientation—are necessary but insufficient without addressing the general institutional structures that exclude all nationalities from effective labor protections. In particular, employer-tie work permit systems (which apply equally to all legally documented workers) and the prohibition on farm workers’ union membership are systemic obstacles that nationality-specific bilateral agreements cannot overcome.

Secondly, the concept of infrastructure shortcomings directly affects labor auditing and supply chain transparency. Malaysia’s current certification frameworks, including the Malaysia Sustainable Palm Oil Program (MSPO) and the multi-sector forced labor indicators developed in response to the U. S. Customs and Border Protection (CBP) freeze order issued to Malaysian palm oil producers in 2020, are designed primarily to detect distorting institutional structures (passport seizures, wage deductions, debt incurred through formal channels). These frameworks lack the capacity to audit labor entirely outside the formal employment system, including undocumented Thai and Indonesian migrant workers whose exploitation is not formally documented. Expanding supply chain auditing to cover informal labor channels requires novel auditing methodologies, including rapid community assessments and collaboration with civil society.

Thirdly, the distinction between freedom of departure and freedom of expression highlights that legalization schemes—which increase official document status and expand the institutional scope for exercising the right to express opinions—are policy mechanisms with the potential to have disproportionate impacts on undocumented migrant workers, who already possess relatively high structural potential for departure. Conversely, for Bangladeshi workers, debt reduction through the enforcement of employer recruitment fees and the imposition of caps on pre-departure expenses is a more impactful intervention, as debt burdens, rather than document status, are the primary constraints on both freedom of departure and freedom of expression.

### 7.7 Limitations of the discussion and directions for future research

The current discussion has several limitations that should be addressed. The proposed theoretical extensions—underlying secondary stratification, out-of-structure capability, infrastructure scarcity, and the distinction between out-of-structure and voice—are based on qualitative evidence from geographically limited frontier contexts and need to be quantitatively tested and iterated in different growing environments (Sabah, Sarawak, and other Peninsular states) before their robust applicability can be established. The cross-sectional design prevents longitudinal analysis of the evolution of vulnerability traits throughout migrants’ life cycles, and future research using panel approaches or a retrospective life-history design would add significant temporal depth to this framework.

The inadequate sample size of workers under the most severe forms of exploitation—those experiencing working conditions akin to forced labor—means that the most extreme levels of vulnerability may have been underestimated in this analysis. Future research should develop methodologies specifically designed to reach this hidden population, perhaps through collaboration with legal aid organizations and survivor-centered participatory research approaches. Furthermore, the gender dimension of vulnerability in plantation work—though mentioned throughout the analysis—deserves a specific comparative examination: female workers performing pesticide spraying, fruit harvesting, and informal farm work face vulnerabilities specific to their gender and migration status, which the current framework primarily addresses through the lens of interconnected factors. Future research should develop a more comprehensive analysis to address this.

## 8 Conclusion

This discussion demonstrates that the working conditions of Thai, Indonesian, and Bangladeshi migrant workers in Malaysian oil palm plantations are not solely a product of employer misconduct or failures in oversight, but rather a result of the systemic interaction of global commodity chain structures, bilateral and national institutional frameworks, social network structures, and individual resources and limitations. The empirical application of the four-dimensional framework extends three fundamental theoretical frameworks: labor market segmentation theory, structural instability, and the politics of liberty, by revealing mechanisms and discrepancies that existing formulas fail to capture, such as sub-level stratification, out-of-structure capability, infrastructure shortcomings, and the fragmentation of freedom of exit and freedom of expression. These contributions advance theoretical understanding of how differential vulnerabilities based on nationality arise across diverse plantation environments and provide a basis for more targeted policy responses. However, the fundamental finding is clear: all three nationality groups—regardless of their level of development in their country of origin, bilateral protection frameworks, or structural out-of-structure capability—are systematically denied the labor rights they are entitled to under Malaysian law and international standards. The differences in their vulnerabilities do not diminish this shared situation. But it points out the specific mechanisms by which similar structural exploitation is transmitted through different channels depending on nationality.

## Data Availability

The datasets presented in this article are not readily available because they consist of qualitative interview and focus group transcripts containing potentially identifying information about undocumented migrant workers. Public deposition would expose participants to risk of immigration enforcement and employer retaliation, and participants did not consent to public data sharing.
